# Fugitivity and *marronage* and the study of sex work

**DOI:** 10.3389/fsoc.2023.1151284

**Published:** 2023-05-19

**Authors:** Julia O'Connell Davidson

**Affiliations:** School of Sociology, Politics and International Studies, University of Bristol, Bristol, United Kingdom

**Keywords:** fugitivity, *marronage*, slavery, prostitution, sex work

## Abstract

Campaigns against female prostitution used slavery as a rhetorical device to characterize the condition of sex workers, and sex work features prominently in contemporary campaigns against “modern slavery”. In both types of campaigning, “the slave” is worked as a symbolic device to represent the abject condition of human beings objectified, controlled by violence or its threat, and stripped of agency and choice. The assumptions and generalizations about prostitution that inform this vision have been extensively critiqued. However, less attention has been paid to the fact that the analogy also rests on a very particular reading of “the slave” and a very partial appeal to histories of Atlantic World slavery. Histories of enslaved people's resistance and flight are entirely overlooked. The latter has recently prompted interest in fugitivity and *marronage* as analytic concepts, albeit concepts that are defined and deployed in different ways by different scholars and activists. This review asks whether and how they might potentially have theoretical purchase with regard to the contemporary experience (both positive and negative) of sex workers.

## Introduction

Those who campaign against female prostitution have long used and continue to deploy slavery as a rhetorical device to characterize the condition of sex workers (Barry, 1984; Jeffreys, 1997; Bindel, 2017). Over the past two decades, female sex workers have also featured prominently in campaigns against so-called “modern slavery” (Bales, 1999; Kara, 2009). Such campaigns often make visual and textual comparisons between what is dubbed “trafficking” or “modern slavery” and the transatlantic slave trade, encouraging audiences to imagine that some or all female sex workers are the modern-day equivalent of enslaved people in the Americas. There many problems with this discourse, not least of which is that Atlantic world slavery was a centuries long and extraordinarily brutal system of domination that was bound up with the development of racial capitalist modernity such that its pernicious consequences still reach into the present (Mills, 1998; Bhattacharyya, 2018; Robinson, 2020). The same cannot be said of sex work *per se*, even if there are cases in which sex work is, or has been, associated with extreme violence. Those who use the prostitution-slavery analogy diminish the enormity of the horror of Atlantic world slavery and “its legacies of anti-black racism” (Beutin, 2017).

A second problem is that they ignore the extensive research literature empirically documenting the fact that, unlike slavery, sex work is often chosen in preference to available alternative livelihood strategies or forms of employment. The same literature shows that there is an immense diversity in terms of the social relations that surround it, and sex work often resembles other service sector jobs and forms of paid work that involve emotional and embodied labor (to mention but a few, O'Connell Davidson, 1998; Sobieszczyk, 2000; Kelly, 2008; Sanders, 2008; Zheng, 2009; Day, 2010; Piscitelli, 2012; Lainez, 2018; Mai, 2018). A third problem is that the representation of prostitution as slavery prompts interventions into the lives of all those who trade sex that are known to lead to extensive and serious rights violations, including “raid and rescue” missions, heavier restrictions on women and children's mobility, forced “rehabilitation,” and further criminalization of already stigmatized and criminalized groups (Soderlund, 2005; Galusca, 2012; Cruz et al., 2019; Walters, 2020; Agarwal, 2021; Shih, 2021; Vanderhurst, 2022).

In the writings of those who describe prostitution as slavery or as a site of “modern slavery,” “the slave” is worked as a symbolic device to represent the abject condition of human beings objectified, controlled by violence or its threat, and stripped of agency and choice. Though the assumptions and generalizations about prostitution that inform this vision have been extensively critiqued, less attention has been paid to the fact that the analogy also rests on a very particular reading of “the slave” and a very partial appeal to histories of Atlantic World slavery, entirely overlooking histories of enslaved people's resistance and flight. The latter has recently prompted interest in fugitivity and *marronage* as analytic concepts, albeit concepts that are defined and deployed in different ways by different scholars and activists. This review considers whether they may potentially have theoretical purchase with regard to the contemporary experience (both positive and negative) of sex workers.

## Revisiting the wrong of slavery: persons and things

In both antislavery discourse on “modern slavery” and radical feminist analyses of prostitution as slavery, slavery's unique and fundamental wrong is taken to be its treatment of human beings as commodities or “things” that can be bought and sold, reducing them to objects of ownership. This fits with longstanding, liberal, legalistic definitions of slavery that assume the enslaved are first and foremost differentiated, controlled and subordinated by their construction (either *de jure* or *de facto*) as objects of property (Allain and Bales, 2012; Bales, 2012). “To determine, in law, a case of slavery, one must look for possession,” and in essence, possession “supposes control over a *person* by another such as a person might control a *thing”* (Allain, 2012, p. 376). This persons/things dichotomy maps onto other key binaries of liberal thought, especially subject/object and agent/victim. The “modern slave” and the “victim of trafficking” are described as stripped of free will and agency, objectified, unable to “walk away” from their exploiter (Bales and Soodalter, 2009). This is echoed in religious and radical feminist campaigns against prostitution, where sex workers appear as voiceless objects, helpless victims, commodities, or “things.”

In stressing the objectification and commodification of the enslaved, today's campaigners forget that “slave” was a status, ascribed by the state in Atlantic world societies (O'Connell Davidson, 2016). It is true this status meant human beings could be treated as *things* or commodities for purposes of accounting and sale, but the enslaved were still *persons* in law in the sense they were deemed legally and morally responsible for any criminal act they committed (Patterson, 1982; Douglass, 1986). Unlike the live-stock to which they were routinely compared, transatlantic slaves were arrested, tried, and punished for committing outlawed acts, which included refusal to submit to the authority of a master or any white person and any effort to escape. Because enslavement did not literally transform human beings into objects, legal ownership of enslaved people did not, in itself, equip the owner with the power to control them “such as a person might control a *thing*.” As human beings, the enslaved retained the capacity to refuse, resist, and run from their captors. For slavery to function as a system of domination, it was necessary to ensure that the consequences of so doing were so terrifying that most enslaved people would choose compliance.

It was the legal ascription of “negative personhood” to the enslaved (Dayan, 2001), and the criminalization and grotesquely violent punishment of their independent action, that walled human beings into the prison of slavery, thereby equipping slaveholders with powers of control. Slavery not only reduced a human being to a piece of property, but also designated “a relation to law, state, and sovereign power; a condition of disfigured personhood, civil incapacitation, and bare life” (Best and Hartman, 2005, p. 10). The dual character of the slave as both person *and* thing was the central ambiguity of New World slavery (Hartman, 1997). The paradox was nowhere more visible than in Fugitive Slave Law in the United States, a body of law that as its name suggests, criminalized enslaved people who attempted to escape. Under Fugitive Slave Law, the runaway slave became criminally liable as a *person* for stealing herself as a *thing* or piece of property (Best, 2004). In law, the enslaved were ephemeral beings: “the slave was always a kind of afterlife, a form of legal being that was neither birthed by law, nor extinguished by law, but nonetheless present in the law” (Han, 2015).

## Fugitive properties

One analytic approach to fugitivity that could be useful to sex work scholars is found in Best (2004) ground-breaking discussion of the fugitivity of the enslaved (human beings who could be legally “owned” as property, yet did not actually become objects, retained a will of their own, could of their own volition flee, or kill, or commit suicide) as a legal conundrum. As Best shows, the question of how to apply modern, capitalist understandings of property ownership as the right to exclude (to have exclusive possession of) to that which is bound up in the human was also difficult in relation to other “things,” such as music, thoughts, ideas. Legal principles developed to accommodate “the slave's coeval status as material property and willing self” were precursors to intellectual property law (2004, p. 17). “Property is a conduit, a relation, not a thing,” Best argues, and forms of law that remain central to understandings of personhood, equality, and race, derive not from definite and determinate objects, “but from fugitive domains of intangible value (i.e., labor, voice, ideas, feelings, principles” (2004, p. 274).

The treatment of human labor power as a commodity presents similar difficulties. The “thing” exchanged across a labor market defies simple possession because it is human, or inheres in a human being who can resist by withholding or absconding with it. It can become fugitive. Likewise, the “property” exchanged by sex workers is ephemeral, inextricably connected to the body and will of the worker and so impossible for any employer or customer to take exclusive possession of. However, where regimes of contract rules have been developed to address this problem for employers (Steinfeld, 1991), sex work is not usually covered, or fully covered, by the body of labor law that regulates employment relations in other sectors (Cruz, 2013). Indeed, it is often fully or partly criminalized.

Where it is unlawful to employ workers to provide sexual services, fugitivity represents a serious problem to any third party who invests in recruiting and employing sex workers. Their outlay on workers' travel/migration expenses, as well as on premises and other accouterments of the trade will be for nothing if the workers abscond, or refuse the hours of work, or are picky about which clients they accept and which services they provide. Many features of the social organization of brothel prostitution are potentially explicable through this lens, from the use of confinement and threat of violence at one extreme, to the practice of charging “house fees” such that workers must pay for the opportunity of employment (O'Connell Davidson, 2006).

Fugitivity is also potentially relevant to the analysis of relations between sex workers and their customers. The “thing” purchased by the customer is elusive. Contracts between sex workers and clients “are virtually always of an incomplete nature,” typically unwritten, only partially orally agreed, and vague as regards the details of the exchange (Adriaenssens and Hendrickx, 2014). They are also unenforceable in many jurisdictions. If the “thing” the customer has paid for escapes him, his options for legal redress are limited. Though sex work is by no means always or necessarily associated with violence, this may help to explain those situations in which customers seek to extract compliance through force or its threat. At the same time, however, the law's failure to make what sex workers sell fully legible as a “thing” or commodity can also be a source of freedom. It enables some to earn a living from a “fugitive domain of intangible value” (the capacity to bring sexual pleasure to another person) whilst evading the systems emplaced to control and exploit other forms of labor.

## Fugitivity as resistance

Early 20^th^ century histories of slavery often reproduced racist assumptions about the “natural” incapacity of Africans and their descendants for freedom, or depicted the enslaved as enfeebled, infantalized, and degraded by their condition (e.g., Elkins, 1959). Against such readings of slavery came histories focusing on ways in which enslaved people actively struggled to transform their own situation (e.g., James, 2001). That approach grew in importance from the 1960s in line both with certain forms of black radical and pan-African politics and with the emergence of New Left social history that focused on history as made by ordinary people and subaltern populations, not simply by powerful elites. Seeing history from “the bottom up” encouraged a preoccupation with enslaved people's resistance and revolt, stimulating studies that produced a new and very different portrait of the enslaved, often one that emphasized their heroism rather than their abjection (Schwartz, 1996). Studies of the many enslaved people who escaped to form “maroon” or “*quilombo*” communities in the hinterlands, or to live as fugitives elsewhere, are a case in point. These histories are often told in such a way as to depict the enslaved as people who, far from having been enfeebled by enslavement, had actively refused it and risked death to pursue and claim their independence (Robinson, 2007; Cheney, 2016).

Writings on prostitution followed a similar trajectory, for political interest in the agency of oppressed groups and their “arts of resistance” (Scott, 1990) also impacted on the study of sex work. Nineteenth and early twentieth century sexologists understood prostitution as a pathological expression of psychological abnormality (Krafft-Ebing, 1998), social reformers and sociologists documented it as a social problem or sometimes a form of deviance that served a necessary social function (Davis, 1937; Sanger, 2022). One strand of feminist thinking had long emphasized prostitution as a social evil borne of patriarchy and poverty, and through the twentieth century to this day, has continued to depict it as a violently destructive and demeaning institution that has devastating effects on “prostituted” women, and harmful consequences for women as a class (Barry, 1996; Jeffreys, 2009). However, from the 1970s, other feminist scholars began to question the history of gender and sexual codes that had produced “the prostitute” as a disturbed, damaged and dangerous figure.

Walkowitz (2017), a leading figure of this new approach, states that feminist historians “created a distinctive body of scholarship” characterized by three things. First, it approached “female prostitution as sexual labor, an integral part of the survivalist strategy of the poor over many centuries;” second it highlighted the negative consequences of policing for women sex workers; third, it “cast doubt on political campaigns, including feminist campaigns that repeatedly ended in legislation and other state actions that marked off sex work from other forms of labour.” Alongside such feminist histories came growing sociological and activist interest in “the ways in which sexual commerce qualifies as work, involves human agency, and may be potentially empowering for workers” (Weitzer, 2007, p. 215). Within this, some scholars and activists have theorized sex workers as resistant subjects (Bell, 1994; Rubin, 2002), “sexual subalterns” (Kapur, 2000), even “the unsung heroes of sexual liberation” (Gallant, 2021).

The idea of sex workers as fugitives resisting the constraints that generally bind people to dominant and oppressive gendered-sexual norms would fit with such analyses, and also perhaps provide insight into the experience and political imaginary of those sex worker activists who do cast sex workers as revolutionaries. But not all sex workers are political activists, and inverting a discourse of victimization to celebrate sex workers *per se* as “the fiercest, sexiest group of freedom fighters the world has ever seen” (Werhun, cited in Gallant, 2021) does not speak to the lived experience described in most research on the topic. Moreover, as with some histories of maroon communities and fugitive slaves, such inversion does not necessarily transcend the liberal conceptual binaries of slavery/freedom, object/subject and victim/agent (Johnson, 2003).

## Fugitivity and escape

Another analytic approach does use fugitivity to question and try to escape liberal binaries. Some scholars and activists are interested in the political possibilities opened up by imagining those who are excluded from the category of Human on the basis of race and gender as fugitives, and conceiving of fugitivity not merely as escape, but as a refusal of that which has refused you (Harney and Moten, 2013). Rather than struggling for “recognition and acknowledgment generated by the system” that relegated “black people, indigenous peoples, queers and poor people” to the “undercommons,” the call is to see beyond that system and its categories “and to access the places we know lie outside its walls” (Halberstram, 2013, p. 6). Or as Emejulu (2022, p. 72) puts it:

“Fugitivity is both the material and discursive movement of Black bodies, minds and souls. The ability to recast ourselves as ungendered–those who shrug off the impossible desire for humanity and deliberately move into an in-between space–would first mean a rejection of a unitary and independent self, as conceived in the liberal democratic imagination.”

Likewise critical of Western philosophical and political traditions that imagine freedom in fixed, static and oppositional terms, political theorist Roberts (2015) draws on Frederick Douglass' idea of movement as “a cornerstone of the human condition and essential to reform and progress... Flight from slavery is a continual process of release from bondage” (2015, p. 41). For Roberts, *marronage* (the process of extricating oneself from slavery) represents “a flight from the negative, subhuman realm of necessity, bondage and unfreedom toward the sphere of positive activity and human freedom.” A focus on the experience of flight, he says, opens up possibilities for a new, dialectical and more relational understanding of freedom, an understanding of “freedom as *marronage*.”

*Marronage* for Roberts, and fugitivity for thinkers like Harney and Moten (2013) and Emejulu (2022), is about escaping the conceptual confines of liberal thought and moving toward a new and different, yet to be imagined, freedom. It serves, in these works, as an abstract and hopeful political concept rather than a descriptor of the historical experience of people who actually lived in maroon communities or as “fugitive slaves.” Indeed, there is much historical evidence to suggest that flight from slavery, even if successful, promised an extremely ambiguous form of “freedom,” one frequently marked by loss, grief, guilt, and loneliness (Brown, 1847; Camp, 2004; Wong, 2009), and little to suggest that escapees from slavery were people who had shrugged off “the impossible desire for humanity” or deliberately moved to “an in-between space.” Nonetheless, as Brown (2009, p. 1249) observes, “If scholars were to emphasize the efforts of the enslaved more than the condition of slavery, we might be able to tell richer stories about how the endeavors of the weakest and most abject have at times reshaped the world.”

Hartman (2019) has recently applied a similar lens to slavery's afterlives, in particular to explore the ways in which impoverished young black women in the early twentieth century United States refused the subordinate existence assigned to them, and rejected the standards of respectability used by sociologists as well as social reformers and state agents to frame them as deviant, criminal or wayward. “Wayward,” Hartman observes, is “related to the family of words: errant, fugitive, recalcitrant, anarchic, willful, reckless, troublesome, riotous, tumultuous, rebellious and wild” (2019, p. 227), and she approaches the wayward lives of these young black women as experiments in the pursuit of freedom, as “the untiring practice of trying to live when you were never meant to survive” (228).

These theoretical approaches to fugitivity and *marronage*, and attention to practices of refusing assigned existences and instead pursuing freedom, could potentially be of value to sex work scholars. in the sense that they focus our attention on people's active and agential efforts to negotiate the spaces between domination and freedom. There is already synergy with Mai (2018) recent analysis of sex work and mobility which addresses the gap between migrants' own understandings of agency and exploitation, and the understandings that inform sexual humanitarian moralities, policies and interventions into the lives of migrant sex workers.

The wayward lives of the young migrants he studied in Europe did not follow a linear path from domination to freedom, but rather shuttled “between ‘errant' and more agentic forms of mobility” (2018, p. 73). Likewise, there are many ethnographies that carefully document the active and agential ways in which people navigate the limited livelihood options available to them, including sex work, without romanticizing sex workers as “freedom fighters” - Shah (2014) study of internal migrant women in Mumbai, Cesnulyte (2020) research on women who sell sex in Mombasa, Lainez's (2018) work on Vietnamese women's sex work migration, to mention but a few. Closer dialogue with theory and debate on enslaved people and their descendants' efforts to negotiate the spaces between domination and freedom might help us move beyond a vision of sex workers as either passive victims of structural forces or heroic revolutionaries, and instead recognize sex work as one of a number of ways in which people attempt to refuse and escape the hand that fate has dealt them, and struggle to remake it.

## Author contributions

The author confirms being the sole contributor of this work and has approved it for publication.
